# Partial anomalous pulmonary venous return and atrial septal defect in adult patients detected with 128-slice multidetector computed tomography

**DOI:** 10.1186/1749-8090-6-126

**Published:** 2011-09-30

**Authors:** Sari Kivistö, Helena Hänninen, Miia Holmström

**Affiliations:** 1 Department of Radiology, University of Helsinki and HUS Radiology (Medical Imaging Center), Haartmaninkatu 4, Helsinki, 00029 HUS, Finland; 2 Department of Cardiology, Helsinki University Central Hospital, Haartmaninkatu 4, Helsinki, 00029 HUS, Finland; 3 Department of Radiology, University of Helsinki and HUS Radiology (Medical Imaging Center), Haartmaninkatu 4, Helsinki, 00029 HUS, Finland

**Keywords:** Partial anomalous pulmonary venous return (PAPVR), Atrial septal defect (ASD), Multidetector computed tomography (MDCT)

## Abstract

The present series describes a group of adults with left-to-right shunts including partial anomalous pulmonary venous return (PAPVR) and/or an atrial septal defect (ASD) evaluated with ECG-gated 128-slice multidetector computed tomography (MDCT). PAPVR is defined as a left-to-right shunt where one or more, but not all, pulmonary veins drain into a systemic vein or the right atrium. PAPVR involving the right upper pulmonary vein can be associated with a sinus venosus ASD. The presence, course, number of anomalous veins and associated cardiovascular defects can be reliably observed by 128-slice MDCT angiography.

## Background

Partial anomalous pulmonary venous return (PAPVR) is defined as a left-to-right shunt where one or more, but not all, pulmonary veins drain into a systemic vein or the right atrium. Anomalous right-sided pulmonary veins can drain into the superior vena cava (SVC), right atrium, inferior vena cava, azygos vein, hepatic vein or portal vein. The connecting sites for anomalous left-sided pulmonary veins can be the left brachiocephalic vein, coronary sinus and hemiazygos vein. PAPVR involving the right upper pulmonary vein can be associated with a sinus venosus atrial septal defect (ASD) located near the SVC orifice [1].

All PAPVRs are left-to-right shunts, but unless more than 50% of the pulmonary flow drains to the right side of the heart clinical manifestations are rare. Dyspnea, fatigue, exercise intolerance, palpitations, syncope, atrial arrhythmias, right heart failure, and pulmonary hypertension may occur [2,3].

The presence, size, and direction of an intracardiac shunt can be noninvasively and accurately evaluated with a peripheral dye dilution technique. The flow ratio of pulmonary to systemic blood flow (P/S) is used clinically to determine the significance of the shunt. The ratio of less than 1. 5 indicates a small left-to-right shunt, 1. 5-1. 9 an intermediate and 2. 0 or more a large left-to-right shunt; the latter two generally require surgical repair to prevent future complications [4]. Although this method is sensitive and accurate in sizing the shunt, the anatomy and location of the shunt remain to be evaluated with other imaging techniques.

PAPVR is usually diagnosed by transthoracic echocardiography (TTE) or transesophageal echocar-diography (TEE) and catheter based angiography [5,6]. However, echocardiography can provide insufficient information, mainly due to its limited acoustic window. Right heart catheterization with pulmonary angiography is an operator-dependent and invasive technique, and it may be difficult to adequately depict, in particular, the anatomy of small accessory and anomalous vessels [2,7,8].

Modern 128-slice multidetector computed tomography (MDCT) scans are accurate in defining ASDs and PAPVR. ECG-gated MDCT enables a non-invasive and rapid image acquisition with high spatial and temporal resolution, optimized contrast bolus timing, and wide anatomic coverage. The presence, course, number of anomalous veins, and associated cardiovascular defects can be reliably observed by MDCT angiography [2,8-11].

The present series describes a group of adults with left-to-right shunts including PAPVR and/or ASD evaluated with ECG-gated 128-slice MDCT.

## Case presentations

All patients were examined with TTE and/or TEE as a part of clinical evaluation. Furthermore, the presence of a left-to-right shunt was confirmed with a peripheral dye dilution technique. P/S ratio of more than 1. 5 was considered significant. Prospectively ECG-gated MDCT angiographies were performed with a 128-slice-scanner (Siemens, AS+). An imaging workstation (Siemens) was used for the interpretation of the volumetric datasets using transverse images complemented by multidimensional images as required.

### Case 1

**Figure 1:**
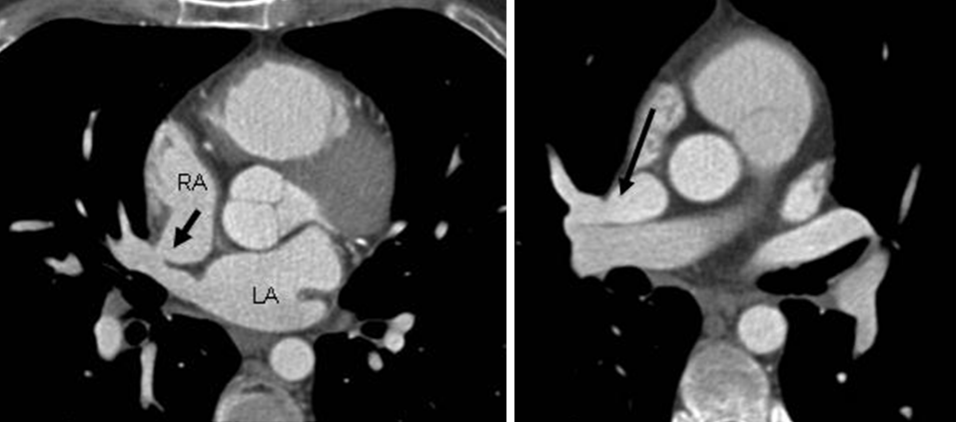
**A superior sinus venosus atrial septal defect (ASD) and partial anomalous pulmonary venous return (PAPVR) on the right side**. A hypertensive male patient with dilatation of the right ventricle documented with echocardiography. Multidetector computed tomography angiography showed a sinus venosus ASD **(a)** and PAPVR from the right upper lobe to the superior vena cava **(b)**. RA = right atrium, LA = left atrium.

A 38-year-old hypertensive male patient with a history of episodes of atrial fibrillation underwent TTE and TEE, which revealed unexplained dilatation of the right ventricle without an ASD or other intracardiac shunt. The peripheral dye dilution curve confirmed a large left-to-right shunt (P/S 2. 8). Axial MDCT images revealed, in addition to a small sinus venosus ASD (Figure 1a), abnormal pulmonary vein drainage from the right upper lobe to the SVC (Figure 1b).

### Case 2

**Figure 2:**
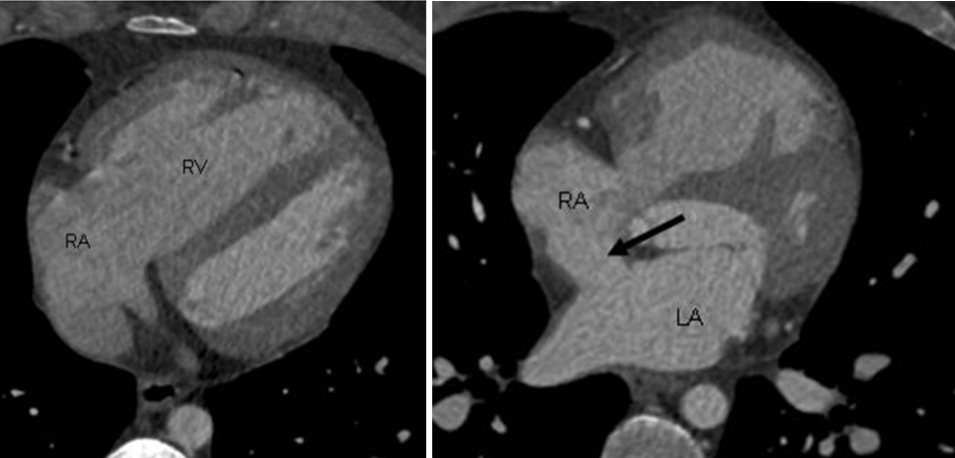
**A dilated right ventricle of the heart and superior sinus venosus atrial septal defect (ASD)**. A male patient with pulmonary hypertension and right-sided volume overload. Multidetector computed tomography documented a dilated right side of the heart **(a)** and a large superior sinus venosus ASD **(b)**, which could not be confirmed with transthoracic echocardiography. RA = right atrium, RV = right ventricle, LA = left atrium.

A 34-year-old male patient with episodes of atrial fibrillation and shortness of breath was diagnosed with pulmonary hypertension and right-sided volume overload. The cause of these findings was not established with TTE. It was not possible to successfully complete TEE. The peripheral dye dilution curve showed a large left-to-right shunt (P/S 2. 2). The patient underwent MDCT to establish the cause of the left-to-right shunt. A series of axial CT scans showed a dilated right ventricle (Figure 2a), a large sinus venosus ASD near the SVC orifice (Figure 2b) and normal pulmonary venous connections.

### Case 3

**Figure 3:**
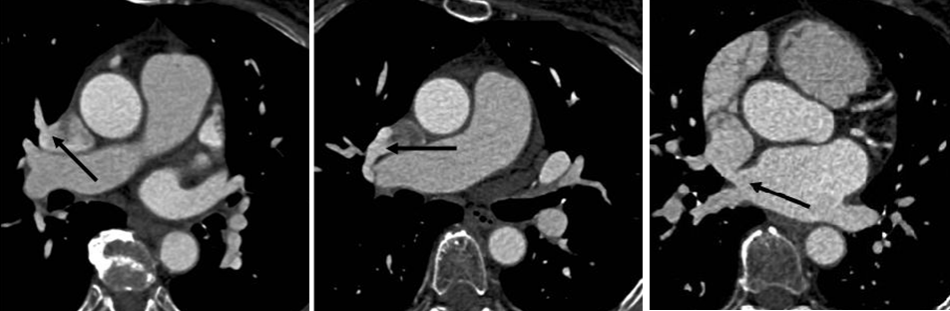
**Anomalous drainage of the right upper pulmonary vein to the superior vena cava and a superior sinus venosus atrial septal defect (ASD)**. A female patient with heart failure and signs of right ventricular overload. Axial images showed partial anomalous pulmonary venous return on the right side **(a-b)** and a small sinus venosus ASD **(c)**.

A 70-year old female patient with heart failure, signs of right ventricular overload, and mild pulmonary hypertension underwent both TEE and catheter based angiography to establish the possible presence of cardiac shunts and the anatomy of the pulmonary veins. On TEE, three shunts were found, two in the atrial septum and one connecting to the SVC. The peripheral dye dilution curve confirmed a large left-to-right shunt (P/S 2. 6). During right heart catheterization, a suspicion of PAPVR on the right side was also documented. The patient was sent to cardiac magnetic resonance imaging (MRI) and later on to MDCT to confirm the findings before surgery. MDCT revealed anomalous drainage of the right upper pulmonary vein to the SVC (Figure 3a, b) and a sinus venosus ASD (Figure 3c).

### Case 4

**Figure 4:**
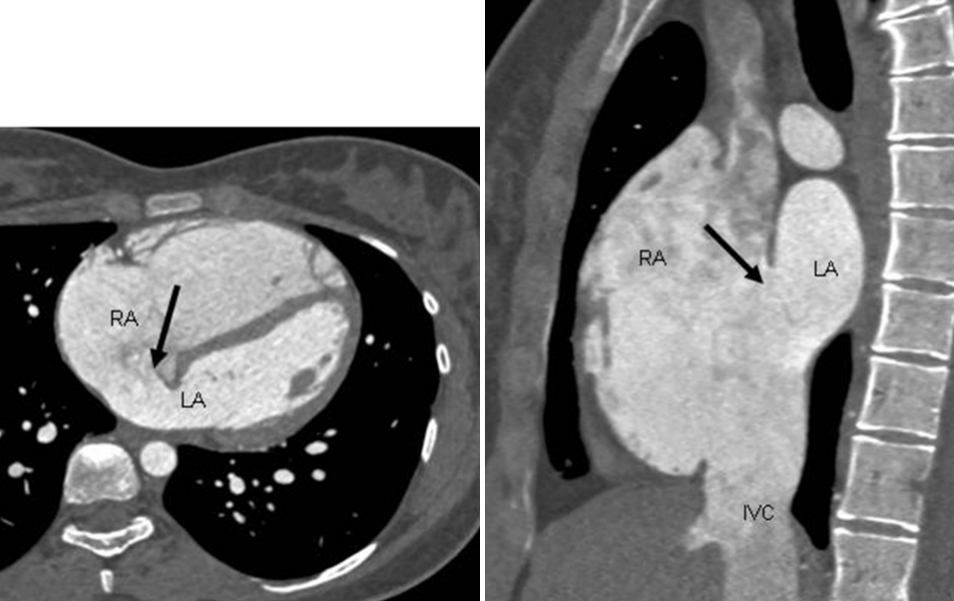
**An inferior sinus venosus atrial septal defect (ASD)**. A young male patient underwent preoperative multidetector computed tomography angiography, which revealed a large multifenestrated inferior sinus venosus ASD in axial **(a)** and sagittal **(b)** images. RA = right atrium, LA = left atrium, IVC = inferior vena cava.

A 20-year-old female patient was sent for cardiac evaluation because of atypical chest pain and atrial fibrillation. TTE showed a dilated right ventricle and a possible ASD, which was confirmed with TEE. The peripheral dye dilution curve showed a large left-to-right shunt (P/S 3. 0-3. 5). The patient was sent to preoperative MDCT to evaluate both the ASD and the anatomy of the pulmonary veins before ASD closure. Axial images (Figure 4a) with sagittal reformats (Figure 4b) confirmed a large multifenestrated inferior sinus venosus ASD, but the pulmonary vein anatomy was normal.

### Case 5

**Figure 5:**
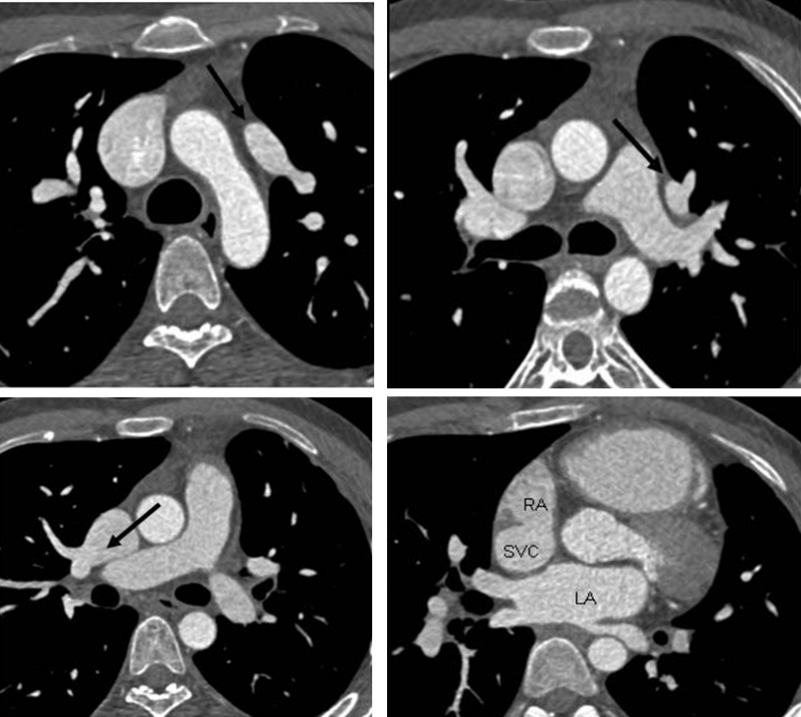
**Partial anomalous pulmonary venous return of both lungs**. An asymptomatic male patient with a dilated right side of the heart documented with thorax x-ray. The left upper lobe vein drains into the brachiocephalic vein **(a-b)**. The right upper lobe vein drains into the superior vena cava **(c)**. Veins of both lower lobes drain normally into the left atrium **(d)**. RA = right atrium, LA = left atrium, SVC = superior vena cava.

A 24-year-old male patient with an enlarged right side of the heart noted in a routine thorax x-ray taken as a part of a physical examination before his military service. TEE did not reveal the cause, and his atrial septum was found to be intact. However, the peripheral dye dilution curve showed a large left-to-right shunt (P/S 2. 4). MDCT showed anomalous pulmonary veins originating from the upper lobe of both lungs (Figure 5a-c). The veins from the right middle lobe and both lower lobes drained normally to the left atrium (Figure 5d).

## Discussion

In recent years modern MDCT and MRI techniques have gained increasing importance in the non-invasive assessment of vascular pathologies of the chest. In our patients, the diagnosis was established with certainty using contrast-enhanced ECG-gated chest MDCT with volume-rendered reconstructions. MDCT provided accurate information of pulmonary vein anatomy and cardiac shunts in patients with right ventricular enlargement.

The isotropic voxel size and good spatial resolution, as compared with other techniques, allow ex-amination of small vessels and shunts with multidimensional reconstructions using advanced workstations [11]. However, there are some drawbacks for the routine use of MDCT [9]. The radiation dose is a concern especially in young patients. However, using the newest technologies the radiation dose for a cardiac structure evaluation is as low as 1-5 mSv. Exposure can be reduced by ECG attenuation techniques that limit exposure during the less informative parts of the cardiac cycle. Gating remains problematic in patients with fast and irregular heart rates. The success of this method is therefore dependent on the correct use of pre-medication, ECG-gating, and special technical protocols. Data-processing of multidimensional images can be time consuming, but 2D- and 3D-images are valuable in the planning of surgery [11].

In some other studies, cardiac MRI has reliably detected and delineated sinus venosus defects and PAPVR. MRI offers several advantages over cardiovascular imaging. MRI does not use ionization radiation and does not necessarily require injection of a contrast medium [7,12]. On the other hand, this method has lower spatial resolution, susceptibility artifacts, increased pixel size, and longer examination times than MDCT [9,10]. In addition, known contraindications to MRI include claustrophobia, pacemakers and metal objects in the body area.

## Conclusions

In our experience ECG-gated MDCT with fast data acquisition and multidimensional reconstructions offers excellent spatial resolution and the possibility to reliably depict intracardiac and pulmonary shunts.

## Consent

Written informed consent was obtained from the patient for publication of this Case report and any accompanying images. All names and social security numbers have been removed from the images.

## List of Abbreviations

PAPVR: partial anomalous pulmonary venous return; MDCT: multidetector computed tomo-graphy; ASD: atrial septal defect; ECG: electrocardiography; SVC: superior vena cava; P/S: pulmonary to systemic blood flow ratio; TTE: transthoracic echocardiography; TEE: transesophageal echocardiography; HU: Hounsfield unit; MRI: magnetic resonance imaging.

## Competing interests

The authors declare that they have no competing interests.

## Authors' contributions

SK, MH: 1) have made substantial contributions to conception and design, acquisition of data, analysis and interpretation of data; 2) have been involved in drafting the manuscript or revising it critically for important intellectual content; and 3) have given final approval of the version to be published.

HH: 1) have been involved in drafting the manuscript and revising it critically for important intel-lectual content; and 2) have given final approval of the version to be published.
